# Supplementary material for: Wastewater Surveillance Captured an Increase in Adenovirus Circulation in Milan (Italy) during the First Quarter of 2022
Source: Viruses. 2022 Oct 26;14(11):2351. doi: 10.3390/v14112351 (PMC9697775; doi:10.3390/v14112351)
Supplement: Supplementary file 1 [file viruses-14-02351-s001.zip › viruses-1951944-supplementary.pdf]

Table S1: AdV-positivity rate by week in respiratory and fecal samples collected from inpatients with respiratory/gastroenteric infections in Fondazione IRCCS Ca' Granda Ospedale Maggiore Policlinico in Milan from week 2021-01 to week 2022-17, and quantification of AdV-DNA (cg/L/day) in urban wastewater samples collected in a wastewater treatment plant in Milan from week 2021-01 to week 2022-17. (ND: not determined.).

ISO week	AdV positive rate (%) in respiratory samples	AdV positive rate (%) in gastrointestinal samples	AdV concentration (cg/L/day)
**2021-01**	3.4%	0.0%	ND
**2021-02**	5.3%	0.0%	6.95E+11
**2021-03**	2.3%	0.0%	1.38E+12
**2021-04**	0.0%	0.0%	2.63E+12
**2021-05**	5.9%	0.0%	1.62E+13
**2021-06**	0.0%	0.0%	6.38E+11
**2021-07**	2.2%	0.0%	7.62E+11
**2021-08**	6.5%	0.0%	2.16E+13
**2021-09**	2.6%	0.0%	2.31E+12
**2021-10**	0.0%	0.0%	1.07E+13
**2021-11**	0.0%	0.0%	1.85E+12
**2021-12**	3.7%	0.0%	1.09E+13
**2021-13**	0.0%	0.0%	2.03E+10
**2021-14**	3.2%	0.0%	1.01E+12
**2021-15**	14.3%	0.0%	2.09E+10
**2021-16**	3.2%	0.0%	ND
**2021-17**	6.1%	0.0%	ND
**2021-18**	6.3%	0.0%	ND
**2021-19**	3.4%	0.0%	ND
**2021-20**	5.3%	0.0%	ND
**2021-21**	10.5%	0.0%	ND
**2021-22**	9.4%	0.0%	ND
**2021-23**	9.1%	0.0%	6.09E+11
**2021-24**	3.4%	0.0%	5.84E+11
**2021-25**	3.2%	0.0%	2.33E+10
**2021-26**	20.7%	0.0%	2.33E+10
**2021-27**	21.1%	0.0%	2.33E+10
**2021-28**	13.8%	0.0%	2.33E+10
**2021-29**	0.0%	0.0%	2.33E+10
**2021-30**	3.2%	0.0%	2.33E+10
**2021-31**	0.0%	0.0%	2.33E+10

**2021-32**	0.0%	0.0%	ND
**2021-33**	6.7%	0.0%	1.92E+14
**2021-34**	7.7%	0.0%	6.13E+13
**2021-35**	0.0%	0.0%	ND
**2021-36**	2.8%	0.0%	ND
**2021-37**	0.0%	0.0%	ND
**2021-38**	2.7%	0.0%	ND
**2021-39**	0.0%	0.0%	ND
**2021-40**	7.5%	0.0%	3.99E+13
**2021-41**	2.2%	0.0%	1.50E+13
**2021-42**	1.9%	15.4%	3.23E+13
**2021-43**	1.5%	5.0%	1.72E+13
**2021-44**	16.4%	50.0%	1.05E+14
**2021-45**	8.1%	6.7%	4.12E+13
**2021-46**	3.4%	0.0%	2.41E+13
**2021-47**	2.6%	0.0%	3.55E+10
**2021-48**	2.3%	0.0%	2.92E+13
**2021-49**	22.7%	0.0%	5.94E+13
**2021-50**	6.5%	0.0%	6.18E+13
**2021-51**	6.0%	0.0%	5.97E+12
**2021-52**	8.3%	0.0%	2.03E+13
**2022-01**	10.5%	22.2%	1.93E+13
**2022-02**	0.0%	0.0%	1.61E+13
**2022-03**	2.0%	0.0%	ND
**2022-04**	7.5%	0.0%	4.84E+13
**2022-05**	3.0%	27.3%	1.32E+13
**2022-06**	0.0%	0.0%	7.76E+13
**2022-07**	4.3%	0.0%	1.77E+14
**2022-08**	14.7%	6.7%	1.20E+14
**2022-09**	7.5%	5.3%	9.79E+13
**2022-10**	12.9%	31.6%	7.99E+14
**2022-11**	4.3%	14.3%	6.56E+14
**2022-12**	7.1%	22.7%	3.29E+14
**2022-13**	9.0%	4.3%	6.39E+13
**2022-14**	8.1%	12.5%	1.36E+14
**2022-15**	7.0%	6.7%	8.87E+13
**2022-16**	5.7%	9.5%	1.79E+14
